# Lung cancer screening with low-dose computed tomography: National expenditures and cost-effectiveness

**DOI:** 10.3389/fpubh.2022.977550

**Published:** 2022-09-29

**Authors:** Xiaohui Zeng, Zhen Zhou, Xia Luo, Qiao Liu

**Affiliations:** ^1^Department of Nuclear Medicine/PET Image Center, The Second Xiangya Hospital of Central South University, Changsha, China; ^2^Menzies Institute for Medical Research, University of Tasmania, Hobart, TAS, Australia; ^3^Department of Pharmacy, The Second Xiangya Hospital of Central South University, Changsha, China

**Keywords:** lung cancer, LDCT, cost-effectiveness, healthcare expenditures, five-year LC mortality, China

## Abstract

**Objective:**

To compare the cost-effectiveness of undertaking low-dose computed tomography (LDCT) screening for early detection of lung cancer (LC) with different frequencies within the healthcare system of China, and estimate the additional national healthcare expenditure and five-year LC mortality associated with different screening frequencies.

**Material and methods:**

A Markov model was established using national LC epidemiological data from the Chinese Center for Disease Control and Prevention, demographic data from the Chinese Statistical Yearbook, and cost and effectiveness data mainly from the Cancer Screening Program in China. The model included thirty sex-specific screening strategies, which were classified by initial screening age (30, 35, 40, 45, and 50), and screening intervals (intervals at single time point, 1, 2, 5, 10, and 20 years). The main model outputs were incremental cost-effectiveness ratios (ICERs), additional national healthcare expenditure and five-year LC mortality.

**Results:**

The ICERs for LDCT screening strategies vs. non-screening strategy ranged from $16,086 per quality-adjusted life-year (QALY) to $3,675,491 per QALY in the male cohort, and from $36,624 per QALY to $5,943,556 per QALY in the female cohort. The annual increment national healthcare expenditures related to LDCT screening were varied from $0.25 to $13.39 billion, with the lower cost in the cohort with older screening ages and lower screening frequencies. More frequent screening with LDCT was associated with a greater reduction in LC death: an annual LDCT screening was linked to an estimated reduction in five-year LC death by 27.27–29.07%, while a one-off screening was linked to a reduction by 5.56–5.83%.

**Conclusion:**

Under a willingness-to-pay (WTP) threshold of three times the Chinese gross domestic product (GDP) per capita (US $37,654), annual screening with an initiating age at 50 was most cost-effective in both male and female cohorts. By taking into account both the national healthcare expenditures and the effect of LDCT screening, our study results support undertaking LDCT screening annually from 50 years old in general populations.

## Introduction

According to the GLOBOCAN statistics, there were approximately 2.2 million new lung cancer (LC) cases and 1.8 million LC-related deaths occurring worldwide in 2020 (1). China remains the hardest-hit country, responsible for more than one-third of the LC cases globally (2). The five-year survival rate of LC patients depends mainly on the clinical stage at the initial diagnosis, with less than 5% of patients at the late-stage and more than 70% at the early-stage (3–5). Although tremendous efforts have been made in LC management over the last decade, the overall five-year survival rate is still unsatisfactory due to delayed diagnosis (5). The improvement of early detection of LC remains a public health priority.

Low-dose computed tomography (LDCT) is known to be effective for the early detection of LC, therefore is beneficial in improving patients prognosis, and reducing their risk of death (6–9). To alleviate the cancer burden at a population-level, the Chinese government has launched two large-scale, population-based nationwide LC screening programs: the Cancer Screening Program in Urban China (canSPUC) and the Rural Cancer Screening Program (RuraCSP). Both canSPUC and ruraCSP use LDCT screening as the main technical means for early detection of LC (10). Based on the positive results from these two programs (11–16), LDCT is recommended as the gold standard for the early detection of LC by the latest Chinese society of clinical oncology (CSCO) (17, 18). However, because of the Chinas large population base (~1.4 billion), there is an exceptionally large population who are eligible for LDCT screening (19). This has imposed a great financial burden to implement a population-based national LC screening program, especially when the eligible individuals are recommended undertaking the screening at an annual basis by Chinese guidelines (20, 21). To find the most reasonable way to implement LC screening, the National LC Screening programme (NLCCP) group has advocated a one-off LDCT screening for the early detection of LC based on their prospective cohort study in 12 cities of eight provinces across China (22). Moreover, although the current Chinese guidelines recommend the age for an initial LDCT screening is 50 years old (20, 23), some Chinese experts proposed to screen individuals from a younger age to maximize the benefit of LDCT screening (13, 24).

The benefit of a population-based screening approach must be weighed against its costs, so as to figure out an evidence-based strategy to best allocate medical resource with maximized cost-effectiveness. To answer this important question, we established Markov model to compare the cost-effectiveness of 30 sex-specific screening strategies classified by five initial screening ages and six screening intervals and their corresponding non-screening strategies within the healthcare system of China. The additional national healthcare expenditure and 5-year LC mortality associated with each screening strategy was estimated.

## Materials and methods

### Study design

**Figure 1:**
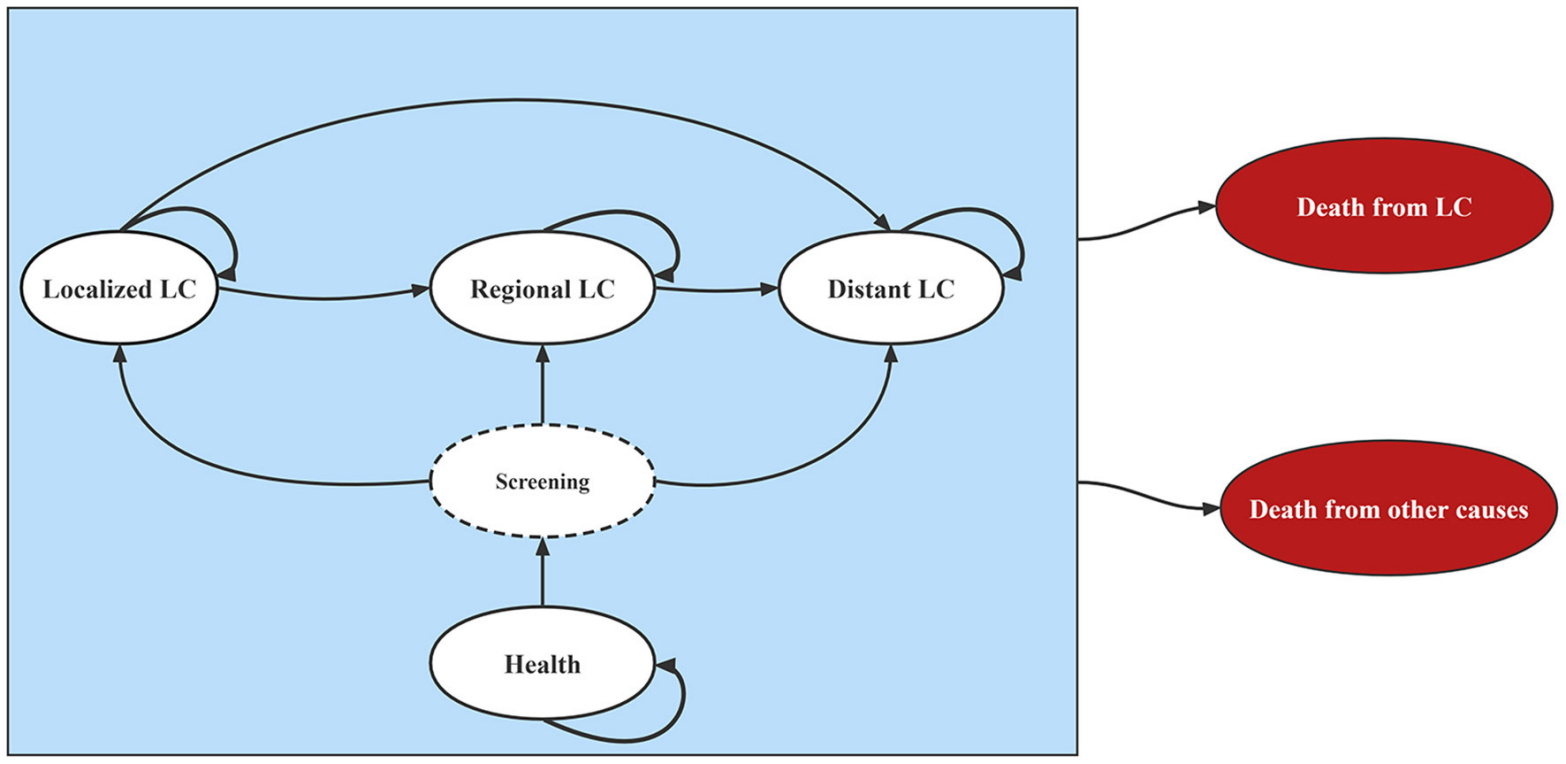
Diagram of Markov model. LC, lung cancer.

A Markov model was designed using TreeAge software (version 2022 R1, https://www.treeage.com/). The Markov model contained six mutually exclusive health states: health, localized LC, regional LC, distant LC, death from LC, and death from other causes (Figure 1). In the model, the TNM stages of LC were reclassified as follows: stages I and II as “localized,” stage III as “regional,” and stage IV as “distant” (25). The arrows in Figure 1 represented the clinical trajectory of LC development. This study was exempted from the ethical review of the Chinese ethics review committee as only published data were used.

Model participants were assumed to be asymptomatic subjects aged 30–79 years with LC who did not have a LC history. The upper age limit was set at 79 years old according to the average life expectancy reported in the Chinese statistical yearbook (CSY) 2021 (26). The estimated population data by sex and age from the CSY 2021, along with the LC incidence, prevalence and mortality data from the Chinese Center for Disease Control and Prevention (CDCP), were used to estimate the number of persons participating in the LDCT screening (Supplementary Tables S1, S2) (26, 27). The number of sex-specific model participants was calculated as the total number of subjects aged 30–79 years without a LC (Supplementary Table S3).

Thirty sex-specific screening strategies classified by five initial screening ages and six screening frequencies, and a non-screening strategies were included in the model. Given the most rapid increase in LC incidence among younger Chinese adults in recent decades (28), this study lowered the initial LDCT screening ages to 30. Five cohorts of subjects who undertook their initial LDCT screening at the age of 30, 35, 40, 45, and 50 years old were compared to determine the optimal initial screening age. Moreover, we constructed six screening frequencies: one-off screening at the initial screening age, annually, every 2 years, every 5 years, every 10 years, and every 20 year, to explore the optimal frequency of undertaking a LDCT screening.

### Transition probabilities

A 1-year transition probability was applied to determine the number of persons in each Markov health state during each Markov cycle. The sex- and age-specific transition probabilities from health to death were identified from the Chinese life table (Supplementary Table S4) (29) and from health to LC were identified from the CDCP reports (Supplementary Table S2) (27). The superiority of LDCT screening over non-screening has been known to be reflected in the relatively high distribution of patients with early-stage LC (6–9). In this study, data of the distribution of patients with localized-, regional- and distant-LC in the screening and non-screening cohorts were derived from a latest comprehensive meta-analysis, which pooled and analyzed all published results of the canSPUC programme and the ruraCSP programme as of February 2021 (30).

The transition probabilities from localized-, regional and distant-LC to death were estimated based on the survival analysis results provided by the Chinese multi-institutional registry (CMIR) (4). The following formula was applied to convert a 5-year mortality rate into a 1-year probability of death: $P_{1-year}=1-exp\left[\frac{\ln\left(1-R_{5-year}\right)}{5}\right]$, where *R*_5−*year*_ denotes the 5-year mortality rate, *P*_1−*year*_denotes the 1-year probability of death (31). The transition probabilities between the three LC health states were derived from an economic evaluation of LDCT screening in 19,770 LC cases collected in the canSPUC programme (32). We set the values of sensitivity and specificity of LDCT screening using data from previous literature (33).

Individuals who participated in the LDCT screening for LC may suffer harm from overdiagnosis (6). Overdiagnosis refers to the diagnosis of a condition that otherwise would not have caused symptoms or death. Based on the long-term follow-up results from the National Lung Screening Trial (NLST), we set the baseline rate of overdiagnosis at 0%, with a range of 0–3% (34). Table 1 summarizes the model parameters regarding transition probabilities.

**Table 1:** Model parameters and assumptions.

**Parameters**	**Values (range)**	**Distribution**	**Reference**
**1-year transition probabilities**			
Health to death	Sex- and age-specific all-cause mortality	/	Chinese life table
Health to LC	Sex- and age-specific LC incidence	/	CDCP
Localized LC to localized LC	0.094000	Beta	CanSPUC
Localized LC to regional LC	0.198000	Beta	CanSPUC
Localized LC to distant LC	0.631836	Beta	CanSPUC
Localized LC to death	0.076164	Beta	CMIR
Regional LC to regional LC	0.389000	Beta	CanSPUC
Regional LC to distant LC	0.418618	Beta	CanSPUC
Regional LC to death	0.192382	Beta	CMIR
Distant LC to distant LC	0.553606	Beta	CanSPUC
Distant LC to death	0.446394	Beta	CMIR
Distribution of LC patients (%)			
Localized LC in screening cohort	91.6	Beta	CanSPUC+RuraCSP
Regional LC in screening cohort	7.6	Beta	CanSPUC+RuraCSP
Distant LC in screening cohort	1.1	Beta	CanSPUC+RuraCSP
Localized LC in the non-screening cohort	21.4	Beta	CanSPUC+RuraCSP
Regional LC in the non-screening cohort	26.3	Beta	CanSPUC+RuraCSP
Distant LC in the non-screening cohort	52.3	Beta	CanSPUC+RuraCSP
Costs (US$)			
Screening	55.03 (44.02–66.03)	Gamma	CanSPUC
Confirmation test for positive cases	180.40 (144.32–216.48)	Gamma	Local hospitals
LC treatments			
Localized LC	10264.91 (8211.93–12317.90)	Gamma	CanSPUC
Regional LC	10795.86 (8636.69–12955.03)	Gamma	CanSPUC
Distant LC	11857.74 (9486.20–14339.29)	Gamma	CanSPUC
Utilities			
Localized LC	0.823 (0.670–0.972)	Beta	(35)
Regional LC	0.772 (0.619–0.921)	Beta	(35)
Distant LC	0.573 (0.420–0.773)	Beta	(35)
Other			
Discount rate (%)	5 (0–8)	Beta	Chinese Guidelines
Overdiagnosis rate (%)	0 (0–3)	Beta	NLST
Sensitivity of LDCT screening (%)	87.70 (71.80–100)	Beta	(33)
Specificity of LDCT screening (%)	90.60 (86.30–91.10)	Beta	(33)

LC, lung cancer; LDCT, Low-dose computed tomography; CDCP, Chinese Center for Disease Control and Prevention; canSPUC, Cancer Screening Program in Urban China; ruraCSP, Rural Cancer Screening Program; CMIR, Chinese multi-institutional registry; NLST, National Lung Screening Trial.

### Cost and effectiveness

We considered the costs for LDCT screening, the confirmation test for positive cases and the treatment for each LC health state (Table 1). According to guidelines, the confirmation test for positive cases consisted of contrast-enhanced CT, positron emission tomography (PET)-CT and biopsy (20, 23). These costs were estimated using data from local hospitals. Costs for the LDCT screening and treatment for each LC health state were collected from the CanSPUC programme (32). All Costs were adjusted in 2020 U.S. dollars based on China's health care consumer price index and the exchange rate of the RMB against the US dollar in 2021 (1 USD = 6.4515 RMB, in 2021) (36).

Effectiveness was measured by quality-adjusted life-years (QALYs), which is calculated as health state utilities weighted life years in the model. The utility value is measured by a score of 0 to 1, where 0 corresponds to death and 1 corresponds to perfect health; specific utility values of distant LC, regional LC and localized LC were obtained from a meta-analysis, which were 0.573, 0.772, and 0.823, respectively (35) (Table 1). Both costs and effectiveness were discounted at 5% annually (37).

### Statistical analysis

Cost-effectiveness between competing strategies was compared by using the incremental cost-effectiveness ratio (ICER), which is defined as the additional costs consumed for each additional QALY. A strategy with an ICER less than $37,654 per QALY (the three times of China's per capita GDP in 2021) was considered cost-effective (36, 37).

The accuracy of the analysis was assessed by deterministic sensitivity analyses (DSA) and probabilistic sensitivity analysis (PSA). DSA was performed separately for individual key parameters to determine their impact on the cost-effectiveness results. During DSA, the test ranges of costs were set from−20% to +20% of the baseline values. The test ranges of utilities (35), over diagnosis rate (34), sensitivity and specificity of LDCT screening (33), were sourced from published literature, and the test range of discounted rate was obtained from Chinese Guidelines (37). PSA was performed for all parameters to evaluate the extent to which the uncertainties of model parameters influenced model results. In PSA, Monte Carlo simulation was carried out through multiple iterations, and the number of iterations was equal to the number of subjects participating in LDCT screening. The uncertainty of model parameters was reflected by the corresponding variation ranges and distributions that are outlined in Table 1.

## Results

### Incremental cost-effectiveness ratio

The results of this cost-effectiveness analysis are summarized in Table 2. Compared with non-screening, the implementation of LDCT screening improved the QALYs by 0.00001–0.02125 at an incremental personal healthcare cost of 26–$1301. The ICERs for LDCT screening strategies vs. non-screening strategy ranged from $16,086 per QALY to $3,675,491 per QALY in the male cohort, and from $36,624 per QALY to $5,943,556 per QALY in the female cohort.

**Table 2:** Cost-effectiveness analysis summary.

**Initial screening age**	**Screening frequency**	**Male**					**Female**				
		**Costs**	**QALY**	**Incremental personal cost^a^**	**Incremental personal QALY^b^**	**Personal ICER ($/QALY)^c^**	**Costs**	**QALY**	**Incremental personal cost^a^**	**Incremental personal QALY^b^**	**Personal ICER ($/QALY)^c^**
/	Non-screening	270	43.05266	/	/	/	129	46.17236	/	/	/
30	One-off	342	43.05268	72	0.00002	3,675,491	201	46.17238	72	0.00001	5,943,556
	Every 20 years	377	43.05451	107	0.00185	57,834	237	46.17322	108	0.00085	126,419
	Every 10 years	437	43.05549	166	0.00283	58,864	298	46.17366	168	0.00129	130,062
	Every 5 years	559	43.05746	289	0.00480	60,192	422	46.17455	292	0.00218	133,908
	Every 2 years	939	43.06433	669	0.01167	57,354	810	46.17779	681	0.00543	125,453
	Every year	1,571	43.07713	1,301	0.02447	53,171	1,452	46.18374	1,322	0.01138	116,205
35	One-off	326	43.05270	56	0.00004	1,422,171	186	46.17239	56	0.00002	2,285,082
	Every 20 years	347	43.05327	77	0.00061	126,449	207	46.17263	77	0.00027	285,300
	Every 10 years	393	43.05464	122	0.00197	62,093	253	46.17325	124	0.00089	139,506
	Every 5 years	487	43.05744	217	0.00478	45,368	350	46.17453	220	0.00217	101,469
	Every 2 years	769	43.06359	499	0.01093	45,663	639	46.17742	510	0.00506	100,774
	Every year	1,244	43.07702	974	0.02436	39,994	1,124	46.18368	995	0.01131	87,967
40	One-off	314	43.05274	44	0.00008	568,259	173	46.17241	44	0.00005	888,447
	Every 20 years	330	43.05364	59	0.00098	60,810	190	46.17280	60	0.00044	137,146
	Every 10 years	365	43.05547	94	0.00281	***33,616***	226	46.17365	96	0.00128	75,089
	Every 5 years	431	43.05740	161	0.00474	***33,900***	293	46.17451	164	0.00215	76,373
	Every 2 years	642	43.06419	371	0.01153	***32,209***	511	46.17770	382	0.00534	71,531
	Every year	990	43.07681	720	0.02415	***29,813***	869	46.18354	739	0.01118	66,138
45	One-off	304	43.05281	34	0.00015	228,027	164	46.17244	34	0.00008	425,637
	Every 20 years	316	43.05403	46	0.00137	***33,507***	176	46.17298	47	0.00062	75,611
	Every 10 years	337	43.05460	66	0.00193	***34,313***	197	46.17323	68	0.00086	78,277
	Every 5 years	387	43.05733	117	0.00466	***25,089***	249	46.17446	120	0.00210	57,155
	Every 2 years	537	43.06332	267	0.01066	***25,082***	406	46.17725	276	0.00489	56,545
	Every year	792	43.07639	522	0.02373	***22,006***	669	46.18327	539	0.01091	49,447
50	One-off	297	43.05297	26	0.00031	85,716	156	46.17251	27	0.00015	179,626
	Every 20 years	305	43.05449	35	0.00183	***19,065***	165	46.17321	36	0.00084	42,714
	Every 10 years	321	43.05539	51	0.00273	***18,559***	181	46.17360	52	0.00123	42,338
	Every 5 years	353	43.05718	83	0.00451	***18,394***	215	46.17438	85	0.00202	42,372
	Every 2 years	462	43.06367	191	0.01100	***17,399***	329	46.17739	200	0.00503	39,685
	Every year	639	43.07558	369	0.02292	***16,086***	513	46.18283	383	0.01046	***36,624***

QALY, quality-adjusted life-year; ICER, incremental cost-effectiveness ratio.

a The incremental personal cost for each screening strategy was calculated as the cost different between the screening strategy and the non-screening strategy.

b The incremental personal QALY for each screening strategy was calculated as the QALY different between the screening strategy and the non-screening strategy.

c ICER values below the willingness-to-pay threshold of $37,654 per QALY are shown in bold and italic font.

**Figure 2:**
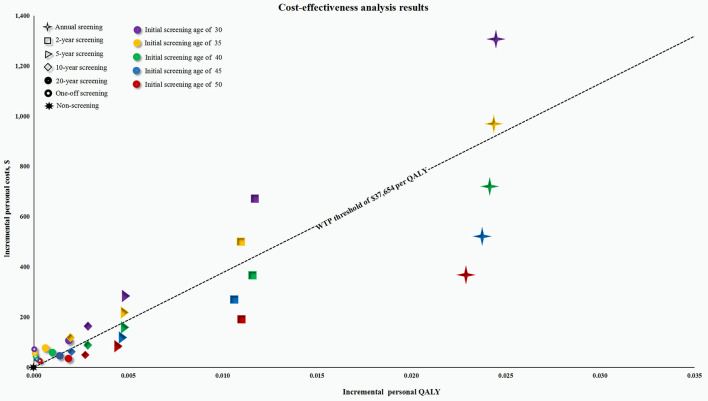
Cost-effectiveness plane for all screening strategies in the male cohort. QALY, quality-adjusted life-year; WTP, willingness-to-pay. The screening strategies represented by the point below the WTP threshold of $37,654 per QALY are considered to be cost-effective; Otherwise they are not cost-effective.

Specifically, the ICERs in the male cohort were below the WTP threshold of $37,654 per QALY, when participants were screened repeatedly from the age of 45 and 50, and every 10 years, every 5 years, every 2 years, and annually from the age of 40 years. After a further comparison between these cost-effective screening strategies, an annual screening strategy with initial screening age at 50 was determined to be the most cost-effective strategy (Figure 2 and Supplementary Table S5). In the female cohort, all the ICERs were above the WTP threshold, except for the participants who were screened annually at the age of 50.

### Sensitivity analysis

Since the model compared up to 30 screening strategies, we only performed sensitivity analysis on the most cost-effective screening strategy (annual screening at initial screening age of 50) vs. the non-screening strategy for both the male and female cohorts. The DSA results demonstrated that in both the male and female cohorts, the discount rate and the overdiagnosis rate had the greatest impact on the ICERs (Supplementary Figure 1). PSA revealed that compared with the non-screening strategy, assuming a WTP threshold of $37,654 per QALY, the cost-effectiveness probability of this LDCT screening strategy was 74.1 % in the male cohort and was 52.9% in the female cohort.

### Annual additional national healthcare expenditure

The annual additional national healthcare expenditures were calculated by dividing the excess national healthcare expenditures in the LDCT screening cohorts relative to the non-screening cohort by the life years. The model showed the annual additional national healthcare expenditures due to LDCT screening ranged from $0.25 to $13.39 billion, which was lower in the cohorts of subjects who undertook a screening at older age and those screened less frequently. When using the same screening strategy, the annual additional national healthcare expenditures were lower in the female cohorts than the male cohort (Supplementary Table S6).

### Five-year LC mortality

We estimated the number of five-year LC death for each LDCT screening strategy by inputting the number of model participants for each initial screening age cohort listed in Supplementary Table S3 through Markov cohort analysis. Our model found that the annual LDCT screening was the most effective in reducing LC death, with five-year LC mortality rate reduced by 27.27–29.07% (Supplementary Table S7).

## Discussion

Using national LC epidemiological data from the CDCP, population data from the CSY, and cost and effectiveness data mainly from the CanSPUC and the RuraCSP, we estimated the cost-effectiveness of the LDCT screening with different frequencies as an early detection method for LC from the perspective of Chinese healthcare system. We also estimated increase in national healthcare expenditure due to the LDCT screening. Results from the model consisting of 30 LDCT screening strategies showed that the LDCT screening strategy became less cost-effective with the decreasing age of initial screening and the screening frequency, especially in the female cohort (Table 2). This can be explained as follows: first, the LC incidence of the younger age cohort was much lower than that of the older age cohort (Supplementary Table S2). This means that the model participants in the younger cohort are less likely to benefit from a LDCT screening. This reason also explains why the LDCT screening is less cost-effective in the female cohort than the male cohort because the higher LC incidence in males than females; second, although more frequent LDCT screening was associated with higher healthcare costs, the greater increase in QALYs made it more cost-effective (Table 2).

Consistent with published randomized controlled trials that demonstrated the effectiveness of LDCT screening in reducing LC mortality (6–9), our model similarly found a lower rate of LC death in the LDCT screening cohorts when compared with the non-screening cohort. As described in Supplementary Table S7, more frequent screening with LDCT was associated with a greater reduction in LC death: an annual LDCT screening was linked to an estimated reduction in five-year LC death by 27.27–29.07%, while a one-off screening was linked to a reduction by 5.56–5.83%. This is mainly due to the fact that more frequent LDCT screening detected more early-stage LC than non-screening, thus reducing more lung cancer deaths. Nevertheless, the reduction in LC mortality in the cohorts with identical screening frequencies seems to be insensitive to the initial screening age. In the analysis, we only considered the five-year LC mortality rate to comply with the Healthy China 2030 programme goal of improving the five-year survival rate of cancer patients.

This study estimated that LDCT screening for LC increased annual national healthcare expenditures by 0.25–$13.39 billion, depending on the initial screening age and the screening frequency. These expenditures account for ~0.7–39.3% of Chinas current annual healthcare expenditures of $34.1 billion for all kinds of cancer treatments (38). The substantial expected budget needed for the implementation of national LDCT screening will impose a great financial burden on Chinese government that has long been engaged in controlling the escalated healthcare costs to ensure the sustainable development of the Universal Medical Insurance System (39). In view of our estimated healthcare budget, it can be expected that the Chinese government will have to cut down the coverage of some expensive healthcare services with unproven clinical benefits and use the savings for the implementation of national LDCT screening for LC. This could be done, for example, through recommending that LDCT screening should be initiated at the age of 50 to contain national healthcare budget, and that LDCT screening should be carried out annually to ensure screening benefits.

Our sensitivity analysis found that the overdiagnosis rate was the most influential parameter that determines the ICERs for repeated screening vs. one-off screening. Overdiagnosis of LDCT screening will undoubtedly lead to increased healthcare expenditures, because individuals who are overdiagnosed will receive unnecessary lung cancer treatments (40). However, the NLST reported an overdiagnosis rate of 18.5% over a mean of 4.5 years of follow-up, while only 3% over a longer follow-up of approximately 9 years, suggesting that long-term follow-up would largely reduce overdiagnosis. Therefore, in our study we set the baseline value of overdiagnosis rate at 0% as the follow-up time is more than 20 years.

Sun C et al. also conducted a cost-effectiveness study on LDCT screening for LC from the perspective of the Chinese healthcare system and estimated that the ICERs of LDCT screening ranged from US 13,056–$15,736 (33). However, our findings cannot be directly compared with theirs for the following reasons. First, we considered the transition between LC states, as well as LC state-specific mortality, while they simply divided LC into early and non-early stages, which may directly influence the estimation of QALYs. Second, we established 30 LDCT screening strategies by varying initial screening ages and screening frequencies, and analyzed them for men and women respectively. Third, they did not assess the budget impact of LDCT screening on national healthcare expenditure.

There are some limitations to this study. First, the model assumed some fixed parameters, such as transition probabilities and distribution of LC patients, the potential heterogeneity of these parameters among screening participants is difficult to identify. Second, we assumed that all individuals who reached the initial screening age would voluntarily participate in the LDCT screening, which may not fully reflect the real-world scenarios; however, the implementation and popularization of national early cancer screening is one of the goals of the Healthy China 2030 programme, so our assumption can provide useful information to Chinese healthcare policymakers; Third, the key parameters used to inform the model were obtained from Chinese settings, which may raise concerns about the generalizability of our conclusions to other nations and regions; however, given the highest distribution of LC cases in China (1, 2), the cost-effectiveness evidence yielded from this study has the potential to help alleviate both national and global cancer disease burdens.

In conclusion, under a WTP threshold of three times the Chinese GDP per capita (US $37,654), annual screening with an initial screening age of 50 was the most cost-effective screening strategy in both the male and female cohorts. To take into account both the national healthcare expenditures and the effect of LDCT screening, results from this study support the implementation of LDCT screening annually for the general population from the age of 50.

## Data availability statement

The original contributions presented in the study are included in the article/Supplementary material, further inquiries can be directed to the corresponding author.

## Author contributions

QL had full access to all of the data in the study and took responsibility for the integrity of the data and the accuracy of the data analysis and statistical analysis. XL and XZ: concept, design, obtained funding, and drafting of the manuscript. XZ, ZZ, XL, and QL: critical revision of the manuscript for important intellectual content and acquisition, analysis, or interpretation of data. All authors contributed to the article and approved the submitted version.

## Funding

This work was supported by the Hunan Provincial Natural Science Foundation [Grant Number 2021JJ80080].

## Conflict of interest

The authors declare that the research was conducted in the absence of any commercial or financial relationships that could be construed as a potential conflict of interest.

## Publisher's note

All claims expressed in this article are solely those of the authors and do not necessarily represent those of their affiliated organizations, or those of the publisher, the editors and the reviewers. Any product that may be evaluated in this article, or claim that may be made by its manufacturer, is not guaranteed or endorsed by the publisher.

## Supplementary material

Supplementary Figure 1 Deterministic sensitivity analysis results for the most cost-effectiveness screening strategy. ICER, incremental cost-effectiveness ratios; QALY, quality-adjusted life-years; WTP, willingness-to-pay; LC, lung cancer; LDCT, low-dose computed tomography.

## References

[1] Sung H Ferlay J Siegel RL Laversanne M Soerjomataram I Jemal A. Global cancer statistics 2020: GLOBOCAN estimates of incidence and mortality worldwide for 36 cancers in 185 countries. CA Cancer J Clin. (2021) 71:209–49. 10.3322/caac.21660 33538338

[2] Cao W Chen HD Yu YW Li N Chen WQ. Changing profiles of cancer burden worldwide and in China: a secondary analysis of the global cancer statistics 2020. Chin Med J. (2021) 134:783–91. 10.1097/CM9.0000000000001474 33734139 PMC8104205

[3] Shao Q Li J Li F Wang S Wang W Liu S. Clinical investigation into the initial diagnosis and treatment of 1,168 lung cancer patients. Oncol Lett. (2015) 9:563–8. 10.3892/ol.2014.2777 25621024 PMC4301476

[4] Liang W Shao W Jiang G Wang Q Liu L Liu D. Chinese multi-institutional registry (CMIR) for resected non-small cell lung cancer: survival analysis of 5,853 cases. J Thorac Dis. (2013) 5: 726–9. 10.3978/j.issn.2072-1439.2013.12.32 24409347 PMC3886697

[5] Zeng H Chen W Zheng R Zhang S Ji JS Zou X. Changing cancer survival in China during 2003-15: a pooled analysis of 17 population-based cancer registries. Lancet Glob Health. (2018) 6:e555–67. 10.1016/S2214-109X(18)30127-X 29653628

[6] The National Lung Screening Trial Research Team. Reduced lung-cancer mortality with low-dose computed tomographic screening. N Engl J Med 365, 395–409. 10.1056/NEJMoa1102873 21714641 PMC4356534

[7] De Koning HJ van der Aalst CM de Jong PA Scholten ET Nackaerts K Heuvelmans MA. Reduced lung-cancer mortality with volume ct screening in a randomized trial. N Engl J Med382. (2020) 503–13. 10.1056/NEJMoa1911793 31995683

[8] Pastorino U Silva M Sestini S Sabia F Boeri M Cantarutti A. Prolonged lung cancer screening reduced 10-year mortality in the MILD trial: new confirmation of lung cancer screening efficacy. Ann Oncol. (2019) 30:1162–9. 10.1093/annonc/mdz117 30937431 PMC6637372

[9] Becker N Motsch E Trotter A Heussel CP Dienemann H Schnabel PA. Lung cancer mortality reduction by LDCT screening-Results from the randomized German LUSI trial. Int J Cancer. (2020) 146:1503–13. 10.1002/ijc.32486 31162856

[10] National Health Commission of the Peoples Republic of China. Measures for the management of urban cancer early diagnosis and treatment projects. Available online at: http://www.nhc.gov.cn/cms-search/xxgk/getManuscriptXxgk.htm?id=56178 (accessed December 15, 2021) (2021).

[11] Guo LW Liu SZ Zhang SK Yang FN Wu Y Zheng LY. Analysis of the efficacy of lung cancer screening in urban areas of Henan Province by low-dose computed tomography from 2013 to 2017. Zhonghua Zhong Liu Za Zhi. (2020) 42:155–9. 10.3760/cma.j.issn.0253-3766.2020.02.013 32135652

[12] Wei MN Su Z Wang JN Gonzalez Mendez MJ Yu XY. Performance of lung cancer screening with low-dose CT in Gejiu, Yunnan: a population-based, screening cohort study. Thorac Cancer. (2020) 11:1224–32. 10.1111/1759-7714.13379 32196998 PMC7180575

[13] Fan L Wang Y Zhou Y Li Q Yang W Wang S. Lung cancer screening with low-dose CT: baseline screening results in Shanghai. Acad Radiol. (2019) 26:1283–91. 10.1016/j.acra.2018.12.002 30554839

[14] Lin Y Ma J Feng J Zhang Q Huang Y. Results of lung cancer screening among urban residents in Kunming. Zhongguo Fei Ai Za Zhi. (2019) 22:413–8. 10.3779/j.issn.1009-3419.2019.07.02 31315779 PMC6712263

[15] Yang L Zhang X Liu S Li HC Li QY Wang N. Lung cancer screening in urban Beijing from 2014 to 2019. Zhonghua Yu Fang Yi Xue Za Zhi. (2021) 55:339–45. 10.3760/cma.j.cn112150-20200817-01126 33730825

[16] Shan W Chen Z Wei D Li M Qian L. Lung cancer screening with low-dose computed tomography at a tertiary hospital in Anhui, China and secondary analysis of trial data. Br J Radiol. (2021) 94:20200438. 10.1259/bjr.20200438 33353400 PMC7934288

[17] Guidelines Working Committee of Chinese society of Clinical Oncology. Guidelines of Chinese Society of Clinical Oncology (CSCO) for Non-Small Cell Lung Cancer [M] 2021 edition. Beijing: People's Medical Publishing House (2021). p. 2021.

[18] Guidelines Working Committee of Chinese society of Clinical Oncology. Guidelines of Chinese Society of Clinical Oncology (CSCO) for Small Cell Lung Cancer [M] 2021 edition. Beijing: People's Medical Publishing House (2021). p. 141.

[19] National Bureau of Statistics of China. China statistical yearbook 2021. Available online at: http://www.stats.gov.cn/english/Statisticaldata/AnnualData/ (accessed November 14, 2021) (2021).

[20] Zhou Q Fan Y Wang Y Qiao Y Wang G Huang Y. China national lung cancer screening guideline with low-dose computed tomography (2018 version). Clin J Lung Cancer. (2018) 21:67–75. 10.3779/j.issn.1009-3419.2018.02.01 29526173 PMC5973012

[21] He J Li N Chen WQ Wu N Shen HB Jiang Y. China guideline for the screening and early detection of lung cancer (2021, Beijing). Clin J Oncol. (2021) 43:243–68. 33752304 10.3760/cma.j.cn112152-20210119-00060

[22] Li N Tan F Chen W Dai M Wang F Shen S. One-off low-dose CT for lung cancer screening in China: a multicentre, population-based, prospective cohort study. Lancet Respir Med. (2022) 10:378–91. 10.1016/S2213-2600(21)00560-9 35276087

[23] He J Li N Chen WQ Wu N Shen HB Jiang Y. China guideline for the screening and early detection of lung cancer (2021, Beijing). Zhonghua Zhong Liu Za Zhi. (2021) 43:243–68. 10.3760/cma.j.cn112152-20210119-00060 33752304

[24] Liu D Huang Y Zhou Q Liu L Che G Lu Y. Pulmonary nodules/lung cancer comprehensive management mode: design and application. Zhongguo Fei Ai Za Zhi. (2020) 23:299–305. 10.3779/j.issn.1009-3419.2020.103.06 32279474 PMC7260385

[25] National Cancer Institute. Surveillance, Epidemiology, and End Results Program. Available online at: http://seer.cancer.gov/ (accessed October 24, 2020) (2020).

[26] National Bureau of Statistics. China Statistical Yearbook 2021. Available online at: http://www.stats.gov.cn/tjsj/ndsj/2021/indexch.htm (accessed February 1, 2022) (2021).

[27] Li X Gao S. Trend analysis of the incidence, morbidity and mortality of lung cancer in China from 1990 to 2019. Chin J Prev Contr Chron Dis. (2021) 29:821–5. 10.16386/j.cjpccd.issn.1004-6194.2021.11.005

[28] Zheng D Chen HQ. Lung cancer screening in China: early-stage lung cancer and minimally invasive surgery 3.0. J Thorac Dis. (2018) 10:S1677–79. 10.21037/jtd.2018.05.206 30034835 PMC6035918

[29] Word Health Organization. Life tables by country. Available online at: https://www.who.int/data/gho/data/indicators/indicator-details/GHO/gho-ghe-life-tables-by-country (accessed May 14, 2021) (2021).

[30] Li Y Du Y Huang Y Zhao Y Sidorenkov G Vonder M. Community-based lung cancer screening by low-dose computed tomography in China: first round results and a meta-analysis. Eur J Radiol. (2021) 144:109988. 10.1016/j.ejrad.2021.109988 34695695

[31] Fleurence RL Hollenbeak CS. Rates and probabilities in economic modelling: transformation, translation and appropriate application. Pharmacoeconomics. (2007) 25:3–6. 10.2165/00019053-200725010-00002 17192114

[32] Wang XL. Lung cancer screening and health economics analysis in Hebei province. Med Health Technol. (2020) 2:1–70.

[33] Sun C Zhang X Guo S Liu Y Zhou L Shi J. Determining cost-effectiveness of lung cancer screening in urban Chinese populations using a state-transition Markov model. BMJ Open. (2021) 11:e046742. 10.1136/bmjopen-2020-046742 34210726 PMC8252866

[34] Aberle DR Adams AM Berg CD Black WC Clapp JD Fagerstrom RM. National lung screening trial research team lung cancer incidence and mortality with extended follow-up in the national lung screening trial. J Thorac Oncol. (2011) 14:1732–42. 10.1016/j.jtho.2019.05.044 31260833 PMC6764895

[35] Sturza J. A review and meta-analysis of utility values for lung cancer. Med Decis Making. (2010) 30:685–93. 10.1177/0272989X10369004 20448248

[36] National Bureau of Statistics. National annual data. Available online at: https://data.stats.gov.cn/easyquery.htm?cn=C01 (accessed January 1, 2022) (2022).

[37] Chinese Pharmaceutical Association. Chinese Guidelines for Pharmacoeconomic Evaluations. Available online at: https://tools.ispor.org/peguidelines/source/China-Guidelines-for-Pharmacoeconomic-Evaluations-2020.pdf (accessed June 1, 2021) (2020).

[38] Zheng RS Shun KX Zhang SW Zeng HM Zhou XN Chen R. Analysis on the prevalence of malignant tumors in China in 2015. Chin J Oncol. (2019) 41:19–28.32398031

[39] Yip W Fu H Chen AT Zhai T Jian W Xu R. 10 years of health-care reform in China: progress and gaps in Universal Health Coverage. Lancet. (2019) 394:1192–204. 10.1016/S0140-6736(19)32136-1 31571602

[40] Yankelevitz DF Henschke CI. Overdiagnosis in lung cancer screening. Transl Lung Cancer Res. (2021) 10:1136–40. 10.21037/tlcr-20-736 33718051 PMC7947395

