# Supplementary figures and images for: Lung cancer screening with low-dose computed tomography: National expenditures and cost-effectiveness
Source: Front Public Health. 2022 Sep 29;10:977550. doi: 10.3389/fpubh.2022.977550 (PMC9558698; doi:10.3389/fpubh.2022.977550)

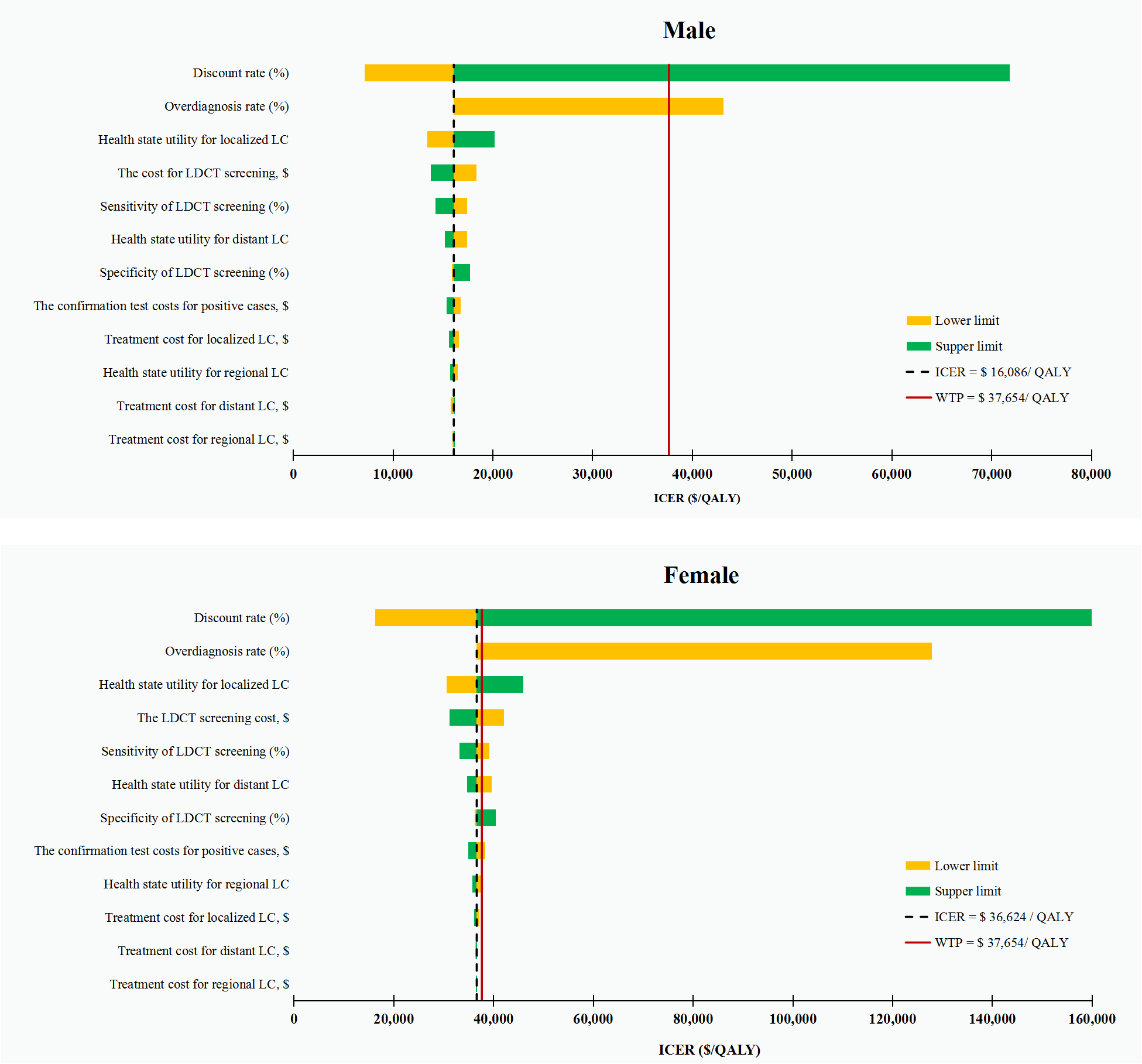

Supplement: Supplementary Figure 1 — Deterministic sensitivity analysis results for the most cost-effectiveness screening strategy. ICER, incremental cost-effectiveness ratios; QALY, quality-adjusted life-years; WTP, willingness-to-pay; LC, lung cancer; LDCT, low-dose computed tomography. [file Image_1.TIF]
