# Supplementary material for: Lung cancer screening with low-dose computed tomography: National expenditures and cost-effectiveness
Source: Front Public Health. 2022 Sep 29;10:977550. doi: 10.3389/fpubh.2022.977550 (PMC9558698; doi:10.3389/fpubh.2022.977550)
Supplement: Supplementary file 2 [file Data_Sheet_1.docx]

Table S1. Sex-specific population in different age groups.

Table S2. Sex-specific incidence, prevalence and mortality of lung cancer in different age groups.

Table S3. The number of sex-specific model participants in each age group.

Table S4. Sex-specific mortality in different age groups.

Table S5. Stepwise ICER comparison of all screening strategies

Table S6. Annual additional national healthcare expenditure.

Table S7. Five-years lung cancer mortality.

Table S1. Gender-specific population in different age groups.

| **Age Group** | **Both sexes** | **Male** | **Female** |
| --- | --- | --- | --- |
| 0-4 years | 77,883,888 | 40,969,331 | 36,914,557 |
| 5-9 years | 90,244,056 | 48,017,458 | 42,226,598 |
| 10-14 years | 85,255,994 | 45,606,790 | 39,649,204 |
| 15-19 years | 72,684,140 | 39,053,343 | 33,630,797 |
| 20-24 years | 74,941,675 | 39,675,995 | 35,265,680 |
| 25-29 years | 91,847,332 | 48,162,270 | 43,685,062 |
| 30-34 years | 124,145,190 | 63,871,808 | 60,273,382 |
| 35-39 years | 99,012,932 | 50,932,037 | 48,080,895 |
| 40-44 years | 92,955,330 | 47,632,694 | 45,322,636 |
| 45-49 years | 114,224,887 | 58,191,686 | 56,033,201 |
| 50-54 years | 121,164,296 | 61,105,470 | 60,058,826 |
| 55-59 years | 101,400,786 | 50,816,026 | 50,584,760 |
| 60-64 years | 73,382,938 | 36,871,125 | 36,511,813 |
| 65-69 years | 74,005,560 | 36,337,923 | 37,667,637 |
| 70-74 years | 49,590,036 | 24,162,733 | 25,427,303 |
| 75-79 years | 31,238,849 | 14,752,433 | 16,486,416 |
| 80+ years | 35,800,835 | 15,257,272 | 20,543,563 |
| Total | 1,409,778,724 | 721,416,394 | 688,362,330 |

Table S2. Sex-specific incidence, prevalence and mortality of lung cancer in different age groups.

| **Age Group** | **LC incidence in total population** | | **LC prevalence in total population** | | **LC mortality in total population** | | **LC mortality in LC population** | |
| --- | --- | --- | --- | --- | --- | --- | --- | --- |
|  | **Male** | **Female** | **Male** | **Female** | **Male** | **Female** | **Male** | **Female** |
| 30-34 years | 0.0000374 | 0.0000229 | 0.0000708 | 0.0000468 | 0.000028 | 0.0000164 | 0.2587800 | 0.3222004 |
| 35-39 years | 0.0000765 | 0.0000470 | 0.0001377 | 0.0000932 | 0.0000599 | 0.0000351 | 0.2796452 | 0.3283442 |
| 40-44 years | 0.0001532 | 0.0000960 | 0.0002433 | 0.0001763 | 0.0001284 | 0.0000759 | 0.0026288 | 0.0010797 |
| 45-49 years | 0.0002981 | 0.0001566 | 0.0005163 | 0.0002982 | 0.0002432 | 0.0001205 | 0.0034191 | 0.0015069 |
| 50-54 years | 0.0006307 | 0.0002925 | 0.0010875 | 0.0005609 | 0.0005134 | 0.0002254 | 0.0053284 | 0.0025489 |
| 55-59 years | 0.0011974 | 0.0004911 | 0.0020562 | 0.0009002 | 0.0009718 | 0.000384 | 0.0082274 | 0.0041111 |
| 60-64 years | 0.0020236 | 0.0008071 | 0.0032336 | 0.0013522 | 0.0016879 | 0.0006577 | 0.0130865 | 0.0068937 |
| 65-69 years | 0.0030138 | 0.0011821 | 0.0043552 | 0.0017433 | 0.0026219 | 0.0010129 | 0.0208797 | 0.0117842 |
| 70-74 years | 0.0044472 | 0.0016944 | 0.0055518 | 0.0021607 | 0.0040813 | 0.0015517 | 0.0376146 | 0.0220304 |
| 75-79 years | 0.0054261 | 0.0021369 | 0.0056667 | 0.0022119 | 0.0053860 | 0.0021382 | 0.0629916 | 0.0384468 |

*LC, lung cancer.*

Table S3. The number of sex-specific participants in each age group.

| **Age Group** | **Total population** | | **Number of persons with LC** | | **Number of persons without LC** | |
| --- | --- | --- | --- | --- | --- | --- |
|  | **Male** | **Female** | **Male** | **Female** | **Male** | **Female** |
| 30-34 years | 63,871,808 | 60,273,382 | 4,522 | 2,821 | 63,867,286 | 60,270,561 |
| 35-39 years | 50,932,037 | 48,080,895 | 7,013 | 4,481 | 50,925,024 | 48,076,414 |
| 40-44 years | 47,632,694 | 45,322,636 | 11,589 | 7,990 | 47,621,105 | 45,314,646 |
| 45-49 years | 58,191,686 | 56,033,201 | 30,044 | 16,709 | 58,161,642 | 56,016,492 |
| 50-54 years | 61,105,470 | 60,058,826 | 66,452 | 33,687 | 61,039,018 | 60,025,139 |
| 55-59 years | 50,816,026 | 50,584,760 | 104,488 | 45,536 | 50,711,538 | 50,539,224 |
| 60-64 years | 36,871,125 | 36,511,813 | 119,226 | 49,371 | 36,751,899 | 36,462,442 |
| 65-69 years | 36,337,923 | 37,667,637 | 158,259 | 65,666 | 36,179,664 | 37,601,971 |
| 70-74 years | 24,162,733 | 25,427,303 | 134,147 | 54,941 | 24,028,586 | 25,372,362 |
| 75-79 years | 14,752,433 | 16,486,416 | 83,598 | 36,466 | 14,668,835 | 16,449,950 |
| Total | 444,673,935 | 436,446,869 | 719,339 | 317,669 | 443,954,596 | 436,129,200 |

*LC, lung cancer.*

*^a^The number of sex-specific persons with LC in each age group was calculated by multiplying the LC prevalence in each age group by the total number of sex-specific persons in this age group, using LC prevalence data from Table S2*

*^c^The number of sex-specific persons without LC in each age group was calculated by subtracting the number of LC from the total number of sex-specific persons in this age group.*

Table S4. Sex-specific mortality in different age groups.

| **Age Group** | **Both sexes** | **Male** | **Female** |
| --- | --- | --- | --- |
| <1 year | 0.006805083 | 0.007220260 | 0.006338840 |
| 1-4 years | 0.000286300 | 0.000302405 | 0.000268089 |
| 5-9 years | 0.000184987 | 0.000195288 | 0.000173127 |
| 10-14 years | 0.000194094 | 0.000241935 | 0.000138345 |
| 15-19 years | 0.000359719 | 0.000481934 | 0.000219306 |
| 20-24 years | 0.000562952 | 0.000791840 | 0.000304858 |
| 25-29 years | 0.000611482 | 0.000875234 | 0.000321813 |
| 30-34 years | 0.000849743 | 0.001224342 | 0.000452289 |
| 35-39 years | 0.001241440 | 0.001773564 | 0.000683142 |
| 40-44 years | 0.001871817 | 0.002628836 | 0.001079694 |
| 45-49 years | 0.002480733 | 0.003419131 | 0.001506936 |
| 50-54 years | 0.003951865 | 0.005328397 | 0.002548890 |
| 55-59 years | 0.006194568 | 0.008227354 | 0.004111115 |
| 60-64 years | 0.009999596 | 0.013086547 | 0.006893700 |
| 65-69 years | 0.016263186 | 0.020879673 | 0.011784175 |
| 70-74 years | 0.029469503 | 0.037614573 | 0.022030414 |
| 75-79 years | 0.049803566 | 0.062991582 | 0.038446795 |
| 80-84 years | 0.091211622 | 0.114511972 | 0.073751188 |
| 85+ years | 0.199643987 | 0.264709858 | 0.163027694 |

Table S5. Stepwise ICER comparison of all screening strategies.

| **Strategy^a^** | **Costs** | **QALY** | **Incremental cost^b^** | **Incremental QALY^c^** | **ICER^d^** |
| --- | --- | --- | --- | --- | --- |
| Non-screening | 270 | 43.05266 | NA | NA | NA |
| 50-oneoff | 297 | 43.05297 | 26 | 0.00031 | 85,716 |
| 45-oneoff | 304 | 43.05281 | 8 | -0.00016 (D) | -46,965 |
| 50-every 20 years | 305 | 43.05449 | 1 | 0.00168 | 537 |
| 40-oneoff | 314 | 43.05274 | 9 | -0.00175 (D) | -5,041 |
| 45-every 20 years | 316 | 43.05403 | 2 | 0.00129 | 1,604 |
| 50-every 10 years | 321 | 43.05539 | 5 | 0.00136 | 3,598 |
| 35-oneoff | 326 | 43.05270 | 5 | -0.00269 (D) | -2,036 |
| 40-every 20 years | 330 | 43.05364 | 3 | 0.00094 | 3,568 |
| 45-every 10 years | 337 | 43.05460 | 7 | 0.00095 | 7,149 |
| 30-oneoff | 342 | 43.05268 | 6 | -0.00191 (D) | -2,981 |
| 35-every 20 years | 347 | 43.05327 | 5 | 0.00059 | 7,977 |
| 50-every 5 years | 353 | 43.05718 | 6 | 0.00391 | 1,618 |
| 40-every 10 years | 365 | 43.05547 | 11 | -0.00171 (D) | -6,636 |
| 30-every 20 years | 377 | 43.05451 | 13 | -0.00096 (D) | -13,065 |
| 45-every 5 years | 387 | 43.05733 | 10 | 0.00281 | 3,589 |
| 35-every 10 years | 393 | 43.05464 | 5 | -0.00269 (D) | -2,036 |
| 40-every 5 years | 431 | 43.05740 | 38 | 0.00277 | 13,808 |
| 30-every 10 years | 437 | 43.05549 | 6 | -0.00191 (D) | -2,981 |
| 50-every 2 years | 462 | 43.06367 | 25 | 0.00818 | 3,064 |
| 35-every 5 years | 487 | 43.05744 | 25 | -0.00622 (D) | -4,080 |
| 45-every 2 years | 537 | 43.06332 | 50 | 0.00588 | 8,585 |
| 30-every 5 years | 559 | 43.05746 | 22 | -0.00586 (D) | -3,683 |
| 50-every year | 639 | 43.07558 | 80 | 0.01812 | 4,405 |
| 40-every 2 years | 642 | 43.06419 | 3 | -0.01139 (D) | -233 |
| 35-every 2 years | 769 | 43.06359 | 128 | -0.00060 (D) | -211,895 |
| 45-every year | 792 | 43.07639 | 23 | 0.01280 | 1,813 |
| 30-every 2 years | 939 | 43.06433 | 147 | -0.01206 (D) | -12,182 |
| 40-every year | 990 | 43.07681 | 51 | 0.01248 | 4,068 |
| 35-every year | 1,244 | 43.07702 | 254 | 0.00021 | 1,185,078 |
| 30-every year | 1,571 | 43.07713 | 327 | 0.00011 | 3,045,285 |
| After excluding dominated strategies: | | | | | |
| Non-screening | 270 | 43.05266 | NA | NA | NA |
| 50-oneoff | 297 | 43.05297 | 26 | 0.00031 | 85,716 |
| 50-every 20 years | 305 | 43.05449 | 8 | 0.00152 | 5,529 |
| 45-every 20 years | 316 | 43.05403 | 11 | -0.00046 (D) | -23,543 |
| 50-every 10 years | 321 | 43.05539 | 5 | 0.00136 | 3,598 |
| 40-every 20 years | 330 | 43.05364 | 9 | -0.00175 (D) | -5,040 |
| 45-every 10 years | 337 | 43.05460 | 7 | 0.00095 | 7,149 |
| 35-every 20 years | 347 | 43.05327 | 10 | -0.00133 (D) | -7,832 |
| 50-every 5 years | 353 | 43.05718 | 6 | 0.00391 | 1,618 |
| 45-every 5 years | 387 | 43.05733 | 34 | 0.00015 | 228,013 |
| 40-every 5 years | 431 | 43.05740 | 44 | 0.00008 | 568,242 |
| 50-every 2 years | 462 | 43.06367 | 31 | 0.00626 | 4,911 |
| 45-every 2 years | 537 | 43.06332 | 76 | -0.00035 (D) | -218,785 |
| 50-every year | 639 | 43.07558 | 101 | 0.01226 | 8,268 |
| 45-every year | 792 | 43.07639 | 153 | 0.00081 | 189,727 |
| 40-every year | 990 | 43.07681 | 198 | 0.00042 | 472,699 |
| 35-every year | 1,244 | 43.07702 | 254 | 0.00021 | 1,185,078 |
| 30-every year | 1,571 | 43.07713 | 327 | 0.00011 | 3,045,285 |
| After excluding dominated strategies: | | | | | |
| Non-screening | 270 | 43.05266 | NA | NA | NA |
| 50-oneoff | 297 | 43.05297 | 26 | 0.00031 | 85,716 |
| 50-every 20 years | 305 | 43.05449 | 8 | 0.00152 | 5,529 |
| 50-every 10 years | 321 | 43.05539 | 16 | 0.00090 | 17,534 |
| 45-every 10 years | 337 | 43.05460 | 16 | -0.00080 (D) | -19,632 |
| 50-every 5 years | 353 | 43.05718 | 17 | 0.00258 | 6,474 |
| 45-every 5 years | 387 | 43.05733 | 34 | 0.00015 | 228,013 |
| 40-every 5 years | 431 | 43.05740 | 44 | 0.00008 | 568,242 |
| 50-every 2 years | 462 | 43.06367 | 31 | 0.00626 | 4,911 |
| 50-every year | 639 | 43.07558 | 177 | 0.01192 | 14,874 |
| 45-every year | 792 | 43.07639 | 153 | 0.00081 | 189,727 |
| 40-every year | 990 | 43.07681 | 198 | 0.00042 | 472,699 |
| 35-every year | 1,244 | 43.07702 | 254 | 0.00021 | 1,185,078 |
| 30-every year | 1,571 | 43.07713 | 327 | 0.00011 | 3,045,285 |
| After excluding dominated strategies: | | | | | |
| Non-screening | 270 | 43.05266 | NA | NA | NA |
| 50-oneoff | 297 | 43.05297 | 26 | 0.00031 | 85,716 (ED) |
| 50-every 20 years | 305 | 43.05449 | 8 | 0.00152 | 5,529 |
| 50-every 10 years | 321 | 43.05539 | 16 | 0.00090 | 17,534 |
| 50-every 5 years | 353 | 43.05718 | 32 | 0.00178 | 18,141 |
| 45-every 5 years | 387 | 43.05733 | 34 | 0.00015 | 228,013 (ED) |
| 40-every 5 years | 431 | 43.05740 | 44 | 0.00008 | 568,242 (ED) |
| 50-every 2 years | 462 | 43.06367 | 31 | 0.00626 | 4,911 |
| 50-every year | 639 | 43.07558 | 177 | 0.01192 | 14,874 |
| 45-every year | 792 | 43.07639 | 153 | 0.00081 | 189,727 (ED) |
| 40-every year | 990 | 43.07681 | 198 | 0.00042 | 472,699 (ED) |
| 35-every year | 1,244 | 43.07702 | 254 | 0.00021 | 1,185,078 (ED) |
| 30-every year | 1,571 | 43.07713 | 327 | 0.00011 | 3,045,285 (ED) |
| After excluding dominated strategies: | | | | | |
| Non-screening | 270 | 43.05266 | NA | NA | NA |
| 50-every 20 years | 305 | 43.05449 | 35 | 0.00183 | 5,529 |
| 50-every 10 years | 321 | 43.05539 | 16 | 0.00090 | 5,530 |
| 50-every 5 years | 353 | 43.05718 | 32 | 0.00178 | 5,531 |
| 50-every 2 years | 462 | 43.06367 | 108 | 0.00649 | 5,532 |
| 50-every year | 639 | 43.07558 | 177 | 0.01192 | 5,533 |

*QALY, quality-adjusted life-year; ICER, incremental cost-effectiveness ratio; NA, not applicable; D, dominated (a strategy is less effective and more costly than a alternative strategy); ED, extended dominated (a strategy is less effective and less cost-effective than a more expensive strategy; or its ICER is high than the willingness-to-pay threshold of $37,654 per quality adjusted life year).*

*^a^For a stepwise ICER comparison, all screening strategies were sorted by cost.*

*^b^The increment cost for each screening strategy was calculated as the cost different between the screening strategy and its previous alternative in the table.*

*^c^A dominated strategies were excluded from the next round of comparison.*

*^c^A extended dominated strategies were excluded from the next round of comparison.*

Table S6. Annual additional national healthcare expenditure.

| **Initial screening age** | **Screening frequency** | **Male** | | | | **Female** | | | |
| --- | --- | --- | --- | --- | --- | --- | --- | --- | --- |
|  |  | **National healthcare expenditure^a^ ($ billions)** | **Additional national healthcare expenditure^b^ ($ billions)** | **Life years** | **Annual additional national healthcare expenditure^c^ ($ billions)** | **National healthcare expenditure^a^ ($ billions)** | **Additional national healthcare expenditure^b^ ($ billions)** | **Life years** | **Annual additional national healthcare expenditure^c^ ($ billions)** |
| NA | Non-screening | 120 | NA | 43.10463 | NA | 56 | NA | 46.19690 | NA |
| 30 | One-off | 152 | 32 | 43.10465 | 0.74 | 88 | 31 | 46.19691 | 0.68 |
|  | Every 20 years | 167 | 47 | 43.10654 | 1.10 | 104 | 47 | 46.19778 | 1.02 |
|  | Every 10 years | 194 | 74 | 43.10757 | 1.71 | 130 | 73 | 46.19825 | 1.59 |
|  | Every 5 years | 248 | 128 | 43.10965 | 2.97 | 184 | 127 | 46.19919 | 2.76 |
|  | Every 2 years | 417 | 297 | 43.11671 | 6.89 | 353 | 297 | 46.20253 | 6.42 |
|  | Every year | 698 | 578 | 43.13004 | **13.39** | 633 | 577 | 46.20875 | 12.48 |
| 35 | One-off | 145 | 25 | 43.10467 | 0.58 | 81 | 25 | 46.19692 | 0.53 |
|  | Every 20 years | 154 | 34 | 43.10527 | 0.79 | 90 | 34 | 46.19719 | 0.73 |
|  | Every 10 years | 174 | 54 | 43.10670 | 1.26 | 110 | 54 | 46.19784 | 1.17 |
|  | Every 5 years | 216 | 96 | 43.10963 | 2.23 | 152 | 96 | 46.19917 | 2.08 |
|  | Every 2 years | 341 | 222 | 43.11601 | 5.14 | 279 | 222 | 46.20218 | 4.81 |
|  | Every year | 552 | 433 | 43.12993 | 10.03 | 490 | 434 | 46.20868 | 9.39 |
| 40 | One-off | 139 | 19 | 43.10471 | 0.45 | 76 | 19 | 46.19695 | 0.42 |
|  | Every 20 years | 146 | 26 | 43.10566 | 0.61 | 83 | 26 | 46.19736 | 0.57 |
|  | Every 10 years | 162 | 42 | 43.10755 | 0.97 | 98 | 42 | 46.19824 | 0.91 |
|  | Every 5 years | 191 | 71 | 43.10959 | 1.65 | 128 | 71 | 46.19915 | 1.55 |
|  | Every 2 years | 285 | 165 | 43.11657 | 3.82 | 223 | 167 | 46.20244 | 3.61 |
|  | Every year | 440 | 320 | 43.12970 | 7.41 | 379 | 322 | 46.20853 | 6.98 |
| 45 | One-off | 135 | 15 | 43.10479 | 0.35 | 71 | 15 | 46.19698 | 0.32 |
|  | Every 20 years | 140 | 20 | 43.10606 | 0.47 | 77 | 20 | 46.19755 | 0.44 |
|  | Every 10 years | 149 | 29 | 43.10666 | 0.68 | 86 | 30 | 46.19781 | 0.64 |
|  | Every 5 years | 172 | 52 | 43.10950 | 1.20 | 109 | 52 | 46.19909 | 1.13 |
|  | Every 2 years | 239 | 119 | 43.11572 | 2.75 | 177 | 120 | 46.20200 | 2.61 |
|  | Every year | 352 | 232 | 43.12926 | 5.37 | 292 | 235 | 46.20824 | 5.09 |
| 50 | One-off | 132 | 12 | 43.10496 | 0.27 | 68 | 12 | 46.19706 | **0.25** |
|  | Every 20 years | 135 | 15 | 43.10652 | 0.36 | 72 | 16 | 46.19777 | 0.34 |
|  | Every 10 years | 142 | 22 | 43.10747 | 0.52 | 79 | 23 | 46.19818 | 0.49 |
|  | Every 5 years | 157 | 37 | 43.10935 | 0.86 | 94 | 37 | 46.19901 | 0.81 |
|  | Every 2 years | 205 | 85 | 43.11601 | 1.97 | 143 | 87 | 46.20211 | 1.88 |
|  | Every year | 284 | 164 | 43.12840 | 3.79 | 224 | 167 | 46.20777 | 3.62 |

*NA, not applicable.*

*^a^The national healthcare expenditure for each screening strategy was calculated by multiplying the personal healthcare costs incurred by the screening strategy and the number of sex-specific model participants.*

*^b^The additional national healthcare expenditure for each screening strategy was calculated as the different in healthcare expenditure between the screening strategy and the non-screening strategy.*

*^c^The annual additional national healthcare expenditures were calculated by dividing the excess national healthcare expenditures in the LDCT screening cohorts relative to the non-screening cohort by the life years.*

Table S7. Five-years lung cancer mortality.

| Initial screening age | Screening frequency | Male | | Female | |
| --- | --- | --- | --- | --- | --- |
|  |  | 5-years LC deaths | 5-years LC deaths reduction (%) | 5-years LC deaths | 5-years LC deaths reduction (%) |
| 30 | Non-screening | 26,985 | / | 16,265 | / |
|  | One-off | 25,431 | 5.76 | 15,317 | 5.83 |
|  | Every 20 years | 25,431 | 5.76 | 15,317 | 5.83 |
|  | Every 10 years | 25,431 | 5.76 | 15,317 | 5.83 |
|  | Every 5 years | 25,431 | 5.76 | 15,317 | 5.83 |
|  | Every 2 years | 22,281 | 17.43 | 13,388 | 17.69 |
|  | Every year | 19,271 | 28.59 | 11,537 | **29.07** |
| 35 | Non-screening | 47,210 | / | 28,761 | / |
|  | One-off | 44,522 | 5.69 | 27,098 | 5.78 |
|  | Every 20 years | 44,522 | 5.69 | 27,098 | 5.78 |
|  | Every 10 years | 44,522 | 5.69 | 27,098 | 5.78 |
|  | Every 5 years | 44,522 | 5.69 | 27,098 | 5.78 |
|  | Every 2 years | 39,090 | 17.20 | 23,720 | 17.53 |
|  | Every year | 33,916 | 28.16 | 20,489 | 28.76 |
| 40 | Non-screening | 81,751 | / | 51,200 | / |
|  | One-off | 77,210 | **5.56** | 48,285 | 5.69 |
|  | Every 20 years | 77,210 | 5.56 | 48,285 | 5.69 |
|  | Every 10 years | 77,210 | 5.56 | 48,285 | 5.69 |
|  | Every 5 years | 77,210 | 5.56 | 48,285 | 5.69 |
|  | Every 2 years | 68,086 | 16.72 | 42,385 | 17.22 |
|  | Every year | 59,460 | **27.27** | 36,769 | 28.19 |
| 45 | Non-screening | 135,859 | / | 71,926 | / |
|  | One-off | 128,198 | 5.64 | 67,798 | 5.74 |
|  | Every 20 years | 128,198 | 5.64 | 67,798 | 5.74 |
|  | Every 10 years | 128,198 | 5.64 | 67,798 | 5.74 |
|  | Every 5 years | 128,198 | 5.64 | 67,798 | 5.74 |
|  | Every 2 years | 112,796 | 16.98 | 59,440 | 17.36 |
|  | Every year | 98,150 | 27.76 | 51,459 | 28.46 |
| 50 | Non-screening | 227,274 | / | 107,519 | / |
|  | One-off | 214,438 | 5.65 | 101,340 | 5.75 |
|  | Every 20 years | 214,438 | 5.65 | 101,340 | 5.75 |
|  | Every 10 years | 214,438 | 5.65 | 101,340 | 5.75 |
|  | Every 5 years | 214,438 | 5.65 | 101,340 | 5.75 |
|  | Every 2 years | 188,751 | 16.95 | 88,859 | 17.36 |
|  | Every year | 164,264 | 27.72 | 76,922 | 28.46 |
